# Acidic pH shock induces the expressions of a wide range of stress-response genes

**DOI:** 10.1186/1471-2164-9-604

**Published:** 2008-12-16

**Authors:** Yoon Jung Kim, Myung Hee Moon, Jae Yang Song, Colin P Smith, Soon-Kwang Hong, Yong Keun Chang

**Affiliations:** 1 Dept. of Chemical and Biomolecular Engineering (The Brain Korea 21 Program), Korea Advanced Institute of Science and Technology, 373-1, Guseong-dong, Yuseong-gu, Daejeon, 305-701, Korea; 2 Functional Genomics Laboratory, School of Biomedical and Molecular Sciences, University of Surrey, Guildford, GU2 7XH, UK; 3 Division of Bioscience and Bioinformatics, Myung-Ji University, San 38-2 Namdong, Yongin, Kyunggido, 449-728, Korea

## Abstract

**Background:**

Environmental signals usually enhance secondary metabolite production in *Streptomycetes *by initiating complex signal transduction system. It is known that different sigma factors respond to different types of stresses, respectively in *Streptomyces *strains, which have a number of unique signal transduction mechanisms depending on the types of environmental shock. In this study, we wanted to know how a pH shock would affect the expression of various sigma factors and shock-related proteins in *S. coelicolor *A3(2).

**Results:**

According to the results of transcriptional and proteomic analyses, the major number of sigma factor genes were upregulated by an acidic pH shock. Well-studied sigma factor genes of *sigH *(heat shock), *sigR *(oxidative stress), *sigB *(osmotic shock), and *hrdD *that play a major role in the secondary metabolism, were all strongly upregulated by the pH shock. A number of heat shock proteins including the DnaK family and chaperones such as GroEL2 were also observed to be upregulated by the pH shock, while their repressor of *hspR *was strongly downregulated. Oxidative stress-related proteins such as thioredoxin, catalase, superoxide dismutase, peroxidase, and osmotic shock-related protein such as vesicle synthases were also upregulated in overall.

**Conclusion:**

From these observations, an acidic pH shock was considered to be one of the strongest stresses to influence a wide range of sigma factors and shock-related proteins including general stress response proteins. The upregulation of the sigma factors and shock proteins already found to be related to actinorhodin biosynthesis was considered to have contributed to enhanced actinorhodin productivity by mediating the pH shock signal to regulators or biosynthesis genes for actinorhodin production.

## Background

Various environmental signals are known to enhance secondary metabolites production in *Streptomycetes *by initiating complex signal transduction system [1]. There have been a number of studies on the application of an environmental stimulus for the enhancement of productivity. These environmental stimuli include heat shock, cold shock, oxidative stress, osmotic stress, acidic sock, alkali shock [2-6].

It is known that different sigma factors respond to different types of stresses, respectively in *Streptomyces *strains, which have a number of unique signal transduction mechanisms depending on the types of environmental shock [1]. Those sigma factors coordinate gene expression in response to various environmental and endogenous signals.

A research group has performed a proteomic study to identify the relationship between proteins expression and environmental stresses, and proteins expression at each phase of cell growth in *S. coelicolor *[7-9]. It was demonstrated that almost all of the shock-related proteins were found in the transient phase just before the stationary phase, implying that various shocks induced the proteins responsible for the initiation of the stationary phase. Based on these findings, it can be deduced that certain types of environmental shock could cause a premature initiation of stationary phase and thus an earlier initiation of secondary metabolites production.

We already observed that an acidic pH shock caused a much earlier initiation of stationary phase and actinorhodin (ACT) production when applied to a surface-grown culture of *Streptomyces coelicolor *A3(2) [10]. In this study, we investigated in transcription and protein levels, the effects of acidic pH shock on the expression of various sigma factors and shock related proteins in *S. coelicolor *A3(2).

## Results and discussion

### Effects of pH Shock on cell growth and actinorhodin production

Actinorhodin is the most abundant product in *S. coelicolor *A3(2). The highest level of actinorhodin production was observed in the pH-shocked culture (designated as PS) in our previous study [10]. In PS, the intracellular actinorhodin of 0.54/(g-cell/L) and extracellular actinorhodin of 1.41/(g-cell/L) were obtained, respectively, which were about 10 times higher than those of the pH non-controlled culture (designated as PNC) and pH controlled culture (designated as PC). Also, we performed transcriptional and proteomic analyses to investigate pH shock effect on the expression of regulatory and biosynthetic genes for actinorhodin production [10]. Four regulators of PkaG, AfsR, AfsS and ActII-ORF4 were observed to be activated by pH shock. In addition, a number of genes known to be associated with actinorhodin biosynthesis were upregulated. In particular, the pathway-specific regulator of *actII-orf4 *having DNA binding activity to *actVI-orf1 *and *actIII-actI *intergenic region is necessary for the initiation of actinorhodin biosynthesis [11]. Such enhanced expression of this regulator is considered to have led to the activation of other genes in the actinorhodin gene cluster. Genes responsible for polyketide synthesis function for actinorhodin production, ketoacylreductase (*actIII*), polyketide beta-ketoacyl synthase alpha/beta subunit (*actI-orf1 *and *-orf2*), actinorhodin polyketide synthase acyl carrier protein (*actI-orf3*), and actinorhodin polyketide synthase bifunctional cyclase/dehydrogenase (*actVII*) [12-14] were also highly upregulated: over 5 fold increased expression compared to that in PC (control). Besides these genes in actinorhodin gene cluster, ActVI-ORF1 and ActVI-ORF3 believed to be responsible for pyran ring closure leading to the formation of the benzoisochromanequinone (BIQ) chromophore [15-17] were upregulated. Among 6 ActVA proteins upregulated, ActVA-ORF4 and ActVA-ORF5 known to play a major role in the reaction of C-6 and C-8 ring hydroxylation in the biosynthesis of aromatic polyketide antibiotics [18] were also upregulated.

How a pH shock-signal can be transferred to the actinorhodin biosynthesis and regulatory genes at the lower level of signal transduction system is still unknown. We, however, considered that signal transduction process initiated by a pH-shock might proceed through sigma factors and shock related proteins at the higher-level. Therefore, the expression patterns of sigma factors and proteins known to be induced by a variety of shocks were examined.

### Expression analysis on a wide range of stress-response genes

**Figure 1:**
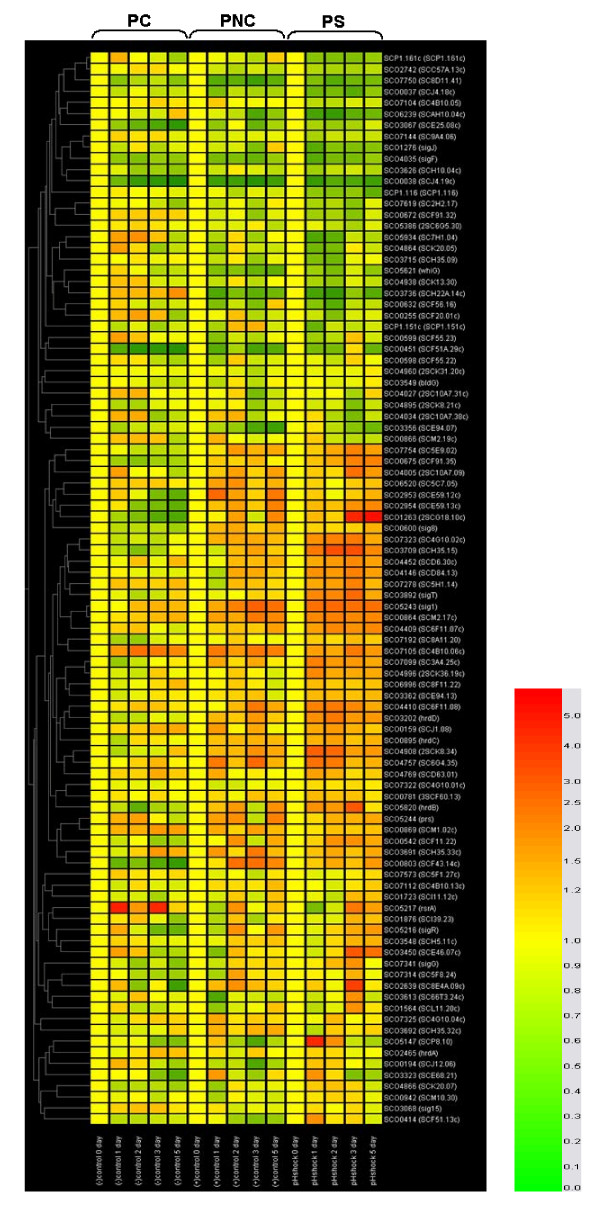
**The result of DNA chip analysis for sigma factors**. Putative RNA polymerase sigma factors (SCO0414; *sigB*, SCO0600; SCO0803; SCO0942; SCO1723; SCO1876; SCO2639; SCO2954; SCO3323; SCO3450; *sigT*, SCO3892; SCO4005; SCO4409; SCO4452; *sigQ*, SCO4908; SCO4996; SCO7099; SCO7192; SCO7314; *sigG*, SCO7341), hypothetical regulatory protein (SCO0542), conserved hypothetical proteins (SCO0675, SCO4757), putative anti sigma factor antagonists (SCO3692, SCO0781, SCO7323, SCO7754), probable ECF-family sigma factors (SCO0864, SCO5147), RNA polymerase principal sigma factors (*hrdC*, SCO0895; *hrdD *SCO3202), putative RNA polymerase ECF sigma factors (SCO1263, SCO3709, SCO4146, SCO4866, SCO4996), putative membrane protein (SCO3362), putative regulatory protein (SCO3691), putative anti anti sigma factor (SCO4410), RNA polymerase sigma factors (*sigR*, SCO5216; *sigH*, SCO5243), major vegetative sigma factor (hrdB, SCO5820), and putative anti-sigma factor (SCO7322) were upregulated in the pH-shocked culture, while putative RNA polymerase sigma factors (SCO0255; SCO2742; SCO5934; SCO6239; SCO7104), putative anti-sigma factor antagonists (SCO0672; SCO4027; SCO7619), probable ECF-family sigma factor (SCO0866), putative integral membrane protein (SCO1632), putative ECF sigma factors (SCO3715; SCO3736; SCO4864; SCO7144), hypothetical protein (SCO4939), RNA polymerase ECF sigma factor (*sigJ*, 1276), SCP1.161c, and SCP1.116 were downregulated in the pH-shocked culture.

**Figure 2:**
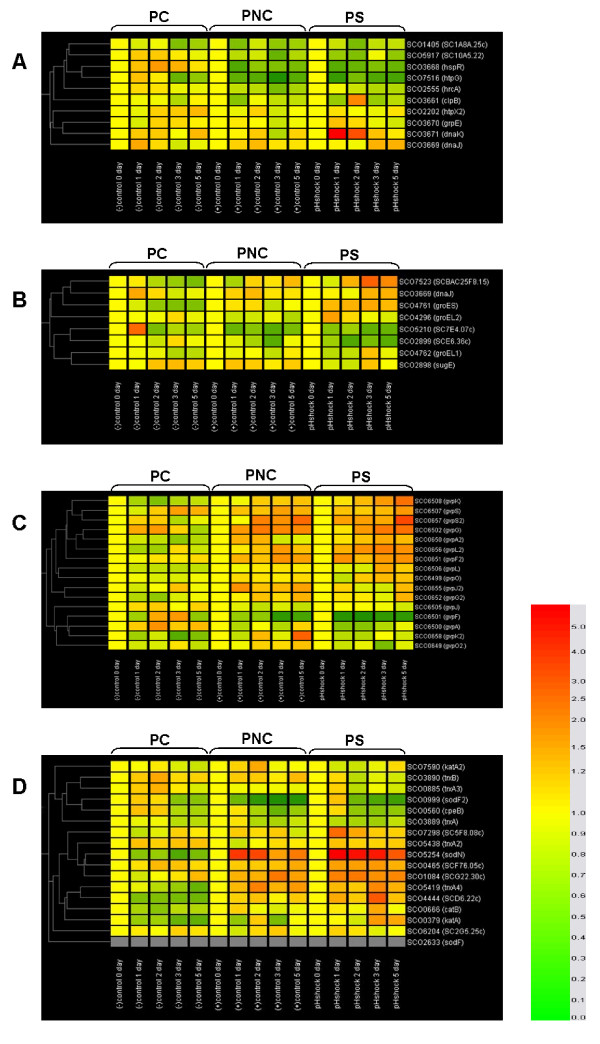
**The result of DNA chip analysis for shock-related proteins**. A) Heat shock proteins. B) Chaperones. C) Osmotic shock proteins. D) Proteins resistant to oxidative stress. Genes coding catalase (*katA*, SCO0379; *catB*, SCO0666), putative gas vesicle synthesis protein (*gvpA2*, SCO0650; *gvpF2*, SCO0651; *gvpG2*, SCO0652; *gvpJ2*, SCO0655; *gvpL2*, SCO0656; *gvpS2*, SCO0657; *gvpO*, SCO6499; *gvpG*, SCO6502; *gvpL*, SCO6506; *gvpS*, SCO6507; *gvpK*, SCO6508;), putative thioredoxin (SCO1084; *trxA4*, SCO5419), ATP-dependent protease ATP-binding subunit (*clpB*, SCO3661), molecular chaperone (*dnaJ*, SCO3669), heat shock protein (*grpE*, SCO3670; *dnaK*, SCO3671), chaperonin 2 (*groEL2*, SCO4296), putative glutathione peroxidase (SCO4444), 10 kD chaperonin cpn10 (*groES*, SCO4761), 60 kD chaperonin cpn60 (*groEL1*, SCO4762), superoxide dismutase (*sodN*, SCO5254), putative DNA-binding protein (SCO6439), putative thioredoxin reductase (SCO7298), putative chaperone (SCO7523) were upregulated with pH shock, while genes coding thioredoxin (*trxA3*, SCO0885), superoxide dismutase (*sodF2*, SCO0999), heat-inducible transcriptional repressor HrcA (SCO2555), molecular chaperone (*sugE*, SCO2898), putative GroES-family molecular chaperone (SCO2899), putative heat shock protein (*hspR*, SCO3668; *htpG*, SCO7516), thioredoxin reductase (NADPH) (*trxB*, SCO3890), conserved hypothetical protein (SCO5917), putative gas vesicle synthesis protein (*gvpA*, SCO6500; *gvpF*, SCO6501) were downregulated.

**Figure 3:**
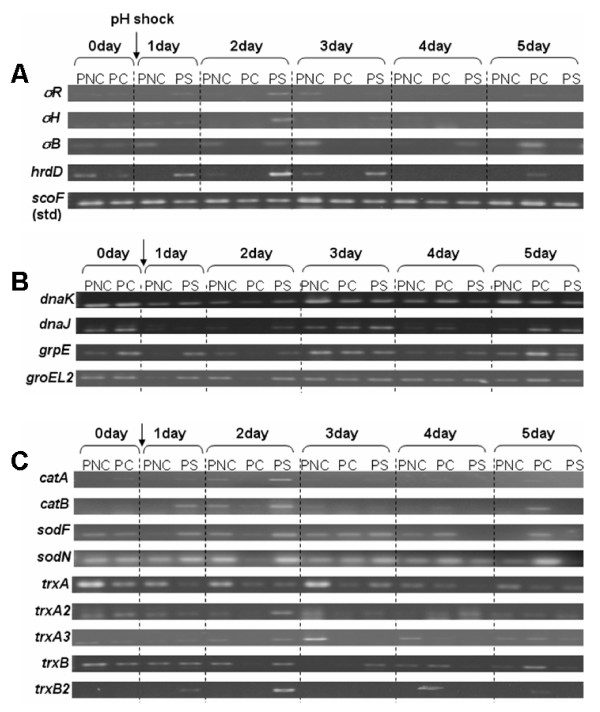
**RT-PCR analysis results of various sigma factors and shock-related proteins**. A) Sigma factors. B) Proteins related to heat shock. C) Proteins related to oxidative stress.

Transcriptional analysis was mainly carried out using DNA chip supplied by Surrey University with triplicate biological repeat samples (Fig. 1 and 2). In addition, to confirm the results from DNA chip analysis, RT-PCR analysis was performed (Fig. 3). A number of sigma factors and shock-related proteins known to be associated with secondary metabolite production, especially actinorhodin production, were observed to be upregulated by an acidic pH shock in overall. In 2-dimensional electrophoresis analysis, three runs of gel electrophoresis were performed, and averaged results were taken. For PNC, PC, and PS, 445, 463, and 324 protein spots were detected, respectively [10]. In MALDI-TOF MS analysis, three proteins associated with sigma factors and gas vesicle synthesis were identified (Table 1). In 1-DE ESI-MS/MS analysis, two chaperone proteins and one protein associated with protection responses were identified for PNC and/or PS as listed in Table 2. None of these proteins were identified in PC.

**Table 1:** 2-DE MALDI-TOF analysis results

**Functional classification**	**Gene bank accession no.**	**Identity**	**Size of protein (kDa/aa)**	**Caculated pI (Expasy)**	**Normalized Vol.**		
					
					**PNC**	**PC**	**PS**
Adaptation	SCO6502	putative gas vesicle synthesis protein (GvpG)	9.7/87	4.2	1.081	0.063	0.939
Sigma factor	SCO6520	putative RNA polymerase sigma factor (Sig K)	30.0/266	5.5	0.013	0.011	2.651
	SCO7112	putative ECF-family RNA polymerase sigma factor	32.8/298	5.9	0.128	0.084	-

**Table 2:** 1-DE ESI-MS/MS analysis results

**Functional classification**	**Gene bank accession no.**	**Identity**	**Size of protein (kDa/aa)**	**Detection**		
				
				**PNC**	**PC**	**PS**
Chaperones	SCO4296	Chaperonin 2 (GroEL2)	56.8/541	no	no	yes
	SCO3671	Chaperone protein dnaK (DnaK)	66.2/618	yes	no	yes
Protection responses	SCO0999	superoxide dismutase	23.6/215	no	no	yes

#### Expression behavior of sigma factors with pH shock

All of the 96 sigma factors, functionally identified or only putative, ever identified in *S. coelicolor *were analyzed for their transcription levels. Among these sigma factors tested, as many as 43 factors were upregulated in the pH-shocked culture as observed by DNA chip analysis, while only 17 factors downregulated (Fig. 1). Among those upregulated, especially, four functionally identified sigma factor genes of *sigT*, *sigU*, *sigB, sigQ *and *hrdB*, and 15 putative sigma factor genes including SCO3709, SCO7323, SCO0542, SCO1263, SCO0675 and SCO7754 were very strongly upregulated by the pH shock. All of the well-known sigma factor genes such as *sigH, sigR, sigG, sigB, hrdD*, and *hrdB *were observed to be upregulated by the pH shock. Besides, many other sigma factor genes of *sigQ, sigT, sigU, bldN *were also upregulated in PS. In overall, their expression pattern in PNC was similar to that in PS, even though their expression levels were much lower than in PS. To confirm these results, RT-PCR was performed on four well-studied sigma factors of *sigR, sigB, sigH *and *hrdD *(Fig. 3A). All of them were expressed in PS and PNC, while none in PC, where pH change was suppressed. Among the strongly upregulated factors, SigH, SigR, and SigB are known to be induced by heat or osmotic, oxidative, and osmotic stress in order. In addition, they are known to have a major role in the secondary metabolite production by activating some major genes related to their biosynthesis [19-22]. SigH is known to play an essential role in the onset of cellular differentiation and antibiotic production [19,23,24]. *S. coelicolor *has *sigB*, which has a high homology with bacillus SigB. SigB is known to control both osmoprotection and differentiation [20]. In addition, Cho *et al*. demonstrated in 2001 that SigB and CatB, catalases were required for osmoprotection and proper differentiation of *S. coelicolor*, and that CatB was under the control of SigB. CatB was also observed to be upregulated in PS [21].

Among them, *hrdD *showed the most sensitive response to pH changes. Their anti-sigma factors were also observed to be induced by the pH-shock (data not shown). This result implied that they must play major roles in the signal transduction system after a pH shock. The amount of the transcript from *hrdD *was reported to increase under sporulation or/and nutrient shift down [25,26]. It is known to be preferentially transcribed by the exponential phase RNA polymerase. HrdD is known to recognize the promoter for *actII-orf4 *regulating the actinorhodin production. Also, The genes of *redD *and *actII-ORF4*, pathway-specific regulators for antibiotic production in *S. coelicolor *A3(2), are reported to be transcribed *in vitro *by an RNA polymerase holoenzyme containing sigma factor, *hrdD *[27]. Therefore, it was considered that the enhancement of actinorhodin production was due to the joint effect of the upregulation of regulatory genes (*pKaG, afsR*, and *afsS*) and actinorhodin pathway specific regulatory gene (*actII*-ORF4) (in our previous study) [10], and the upregulation of *hrdD *(in this study). In addition, two putative RNA polymerase sigma factors of SigK and SCO7112 were observed to be upregulated by the pH shock in 2-DE MALDI-TOF analysis (Table 1). Of these two, SigK having a homology with SigB is known to be induced transiently by heat and salt stress [28].

The analysis results and published information on these sigma factors give a good insight into how a pH shock could enhance actinorhodin production.

#### Expression behavior of heat shock-related proteins

DnaK family, the well-known heat shock proteins of *dnaK*, *dnaJ*, *clpB*, and *grpE *[29-31] were observed to be upregulated by the pH shock in the transcriptional level. In the proteomic analysis also, DnaK was detected only in PNC and PS (Table 2).

In DNA chip analysis, the genes coding well-known heat shock proteins of *dnaK*, *clpB*, and *grpE *were observed to be upregulated in PS (Fig. 2A). In particular, the transcription of *dnaK *steeply increased after pH shock. It showed about 6.2-fold increased expression level one day after the pH shock. On the contrary, the gene coding the repressor of the DnaK family *hspR*, identified by Bucca et al. [31] for the first time in 1995, was strongly downregulated by the pH shock. In RT-PCR analysis, they were expressed one day after the pH shock in PS, while its expression was somewhat delayed in PNC to be observed 2 days after the pH shock. It was not expressed in PC (Fig. 3B).

Among eight chaperone genes in *S. coelicolor *A3(2), five of them (*dnaJ*, *groEL2*, *groES*, *groEL*, and SCO7523) were upregulated. Especially, *groES*, *groEL2*, and SCO7523 were strongly upregulated by the pH shock (Fig. 2B). Chaperonin 2 (or GroEL2) and a chaperone protein of DnaK were also detected in PS by 1-DE ESI-MS/MS analysis (Table 2). Especially, GroEL2 is known to be induced either by heat shock or by undefined physiological general stress signals [32,33]. It is associated with specialized metabolic functions including stationary phase metabolism, the stringent response, protein secretion, and cellular differentiation, playing a special role in the assembly of multienzyme complexes that synthesize secondary metabolites containing peptide or polyketide bonds [34]. Hence, we deduced that GroEL2's induction by the pH shock have, at least partially, contributed to the enhanced actinorhodin biosynthesis.

#### Expression behavior of oxidative stress-related proteins

The expressions of oxidative stress related proteins of catalase, superoxide dismutase, peroxidase, and thioredoxin system were investigated. (Fig. 3C and 2D, and Table 2). In the transcriptional level, genes of *catA, catB, sodN *and *sodF *were upregulated by the pH shock. Among them, *sodN *was much more strongly upregulated than the others. Unfortunately, in DNA chip analysis, another superoxide dismutase gene, *sodF *could not be analyzed because of defects on the DNA chip used. These dismutases and catalases are representative antioxidant enzyme groups in the primary and secondary metabolisms, respectively. Superoxide dismutase (Sod) transforms preferentially reactive oxygen species, especially oxygen radicals, to H_2_O_2 _[35,36], and then hydrogen peroxide generated in the previous step is changed to nontoxic H_2_O by catalase. Catalases of *catA *and *catB *play major roles in the first and secondary metabolism in *S. coelicolor *[37]. Also, Sod was detected in protein level only in PS as shown in Table 2, which is consistent with previous result of transcriptional analysis.

Peroxidases and thioredoxin systems were also observed to be activated in overall by the pH shock. Especially, the putative thioredoxin genes, SCO1084 and *trxA4*, and the putative thioredoxin reductase gene, SCO7298 were strongly upregulated. It is reported that the thioredoxin system begins to work actively under the influence of SigR when oxidative stress exists to generate NADP^+ ^relieving this stress [38-40].

#### Expression behavior of osmotic shock-related proteins

Expression profiles of sixteen gas vesicle synthesis protein genes were investigated for the first time in transcriptional level in this study (Fig. 2C). It has been speculated that gas vesicles might serve a function responsive to osmotic stress [20]. Among those, eleven genes were upregulated in PS, while only two genes were downregulated. The protein of GvpG was also detected in PNC and PS through the 2-DE MALDI-TOF analysis (Table 1). This result might be not sufficient to provide a direct evidence on the relationship between *gvp *upregulation by acidic pH shock and actinorhodin biosynthesis, since the function of *gvp *genes is still not clear. It is, however, considered that these genes might be candidates to mediate a pH shock signal to the genes related to actinorhodin biosynthesis.

## Conclusion

Based on these combined observations, an acidic pH shock was considered to be one of the strongest stresses to influence a wide range of sigma factors and shock-related proteins including general stress response proteins. The upregulation of the sigma factors and shock proteins, especially HrdD, SigH, SigR, SigB and GroEL2, already found to be associated in actinorhodin biosynthesis were considered to have contributed to the enhanced actinorhodin productivity with the pH shock, mediating the pH shock signal to regulators or biosynthesis genes for actinorhodin production.

## Methods

### Strain, media and culture conditions

**Figure 4:**
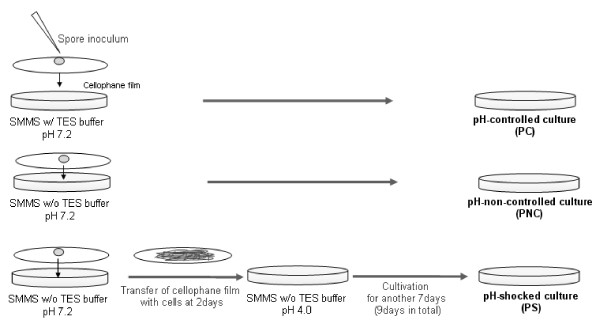
**Experimental design for solid culture to investigate effects of acidic pH shock**.

*S. coelicolor *A3(2) M145 (ATCC BAA471) was grown on a cellophane film placed on supplemented minimal medium, solid (SMMS) at 28°C [38]. A SMMS *with no TES buffer *was used to eliminate the buffering effect and thus to allow pH changes during the culture (Fig. 4). The initial pH was 7.2. Cells were cultivated for 2 days before being transferred to a new SMMS plate with a pH of 4. Just before the transfer, the pH of the medium was about 5.3. The transferred cells were incubated for another 7 days (9 days in total). This pH-shocked culture was designated as PS. The culture with no transfer to a new medium was designated the pH-non-controlled culture (PNC). For the pH-controlled culture (PC), cells were cultivated on the normal SMMS medium with TES buffer to suppress pH changes. The pH of the solid media was measured by using TEST PAPER (Toyo Roshi Kaisha, Japan). A spore stock in 20% glycerol stored at -70°C was used for inoculation.

### Analysis

#### Cell and actinorhodin concentrations

Cell concentration was measured in dry cell weight (DCW). Cells collected off the cellophane film were washed with a phosphate buffer. The washed cells were dried at 80°C for 24 hrs, and then weighed at room temperature. The intracellular and extracellular amounts of actinorhodin produced were separately measured following the procedures previously reported [10,33,34].

### Transcriptomic analyses

An RNeasy Midi kit (Qiagen) was used for RNA isolation according to the manufacturer's instructions. The total RNA was quantified using a NanoDrop ND-1000 (Nanodrop, USA). RNA integrity was assessed using a Bioanalyzer (Agilent Technologies).

The methods of DNA microarray analysis used are detailed at  and in the previous report [10]. Briefly, for RNA labeling, 15 μg of total RNA and 1.7 μl of random primer (Invitrogen) were mixed and incubated for 10 min at 70°C, snap cooled on ice and then 6 μl of 5× First strand buffer, 3 μl of 100 mM DTT, 0.6 μl of dNTP (25 mM each dA/G/TTP, 10 mM dCTP), 2 μl of a Super Script II (Invitrogen) and 1.5 μl of Cy3-dCTP (Amersham Biosciences) were added to make a final volume of 30 μl. The mixture was incubated for 10 min at 25°C in the dark and further incubation was done for 120 min at 42°C in the dark. Ten microliter of 1 N NaOH was added to the incubated mixture. After incubation for 10 min at 70°C, 10 μl of 1 N HCl was added for RNA denaturation.

For genomic DNA labeling, 3.5 μg of genomic DNA, 1 μl of random primer (Invitrogen) and distilled water were mixed to make a final volume of 41.5 μl. The mixture was heated to 95°C for 5 min and snap cooled on ice and then added with 5 μl of 10× Klenow buffer, 1 μl of dNTP (5 mM each dA/G/TTP, 2 mM dCTP), 1.5 μl of Cy5-dCTP (Amersham Bioscience), and 1 μl of Klenow fragment (New England Biolabs, UK). Oligonucleotide DNA microarrays representing 98% of *S. coelicolor *ORF's (fabricated in the Functional Genomics Laboratory, University of Surrey, UK) were used . Hybridization was carried out using a Pronto! Universal Hybridization kit (Corning, USA) according to the supplier's instruction. Equal amounts of Cy3-labeled and Cy5-labeled samples were mixed and dried completely using a vacuum centrifuge. The dried sample was redissolved in 40 μl of a Pronto! Universal Hybridization Solution for long oligonucleotides. The resulting hybridization solution was heated to 95°C for 5 min and applied to a microarray. The hybridized microarray was incubated for 16 hrs at 42°C and then scanned with an Affymetrix 428 scanner (Affymetrix, USA). The 16 bit-TIFF image obtained was analyzed using BlueFuse 2.0 (BlueGnome, Cambridge, UK) and GeneSpring GX 7.3.1 (Agilent Technologies). RT-PCR was carried out by using the primer pairs listed in Table 3. Transcript detection analysis was carried out by using a SUPERSCRIPT One-Step RT-PCR kit (Invitrogen) with 0.25 μg of total RNA as template. ScoF, a cold shock protein of *S. coelicolor*, was used as the internal standard (Fig. 3).

**Table 3:** Oligonucleotide primers used for RT-PCR

**Genes**	**Oligonucleotides**	**Product length**	**Genes**	**Oligonucleotides**	**Product length**
*sigR*	F: 5'-cgt cta tcc tcc gat tcg agt gag-3' R: 5'-gcc ctc gac gtc cgc cag ata cac-3'	568 bp	*catA*	F: 5'-gcc tcc tac cgg cac atg cac gg-3' R: 5'-ctg gtt gcc gtc gac gcg cat gg-3'	500 bp
*sigH*	F: 5'-gtg tcc cag atc gca ggc gag ccc-3' R: 5'-cac gtc ggc ccg gag gag acc ttg-3'	414 bp	*catB*	F: 5'-gac gtg gtg cac gcg gcg aag cc-3' R: 5'-gca gcg ggt cgt cgg tga cgt cg-3'	500 bp
*sigB*	F: 5'-gct cac cgt gct gga gga ggg cac-3' R: 5'-ccg atg aag tcg gcg agc gcc gac-3'	504 bp	*sodF*	F: 5'-gcc gga ggg gat ccg cca tgt cc-3' R: 5'-ggt gga gcc ctg gcc gac gtt gc-3'	500 bp
*hrdD*	F: 5'-ctt ctc gat ctg gcg gat gcg ctc-3' R: 5'-gtc gag aag ttc gac tac cgc aag g-3'	519 bp	*sodN*	F: 5'-ccc gcc tgt ttg ccc cca agg tc-3' R: 5'-cga tgg tgc acc gat cgc cgg-3'	500 bp
*scoF*	F: 5'-gtg ccc gag gtg acg gac gcg-3' R: 5'-tca gac cac gtc ggc cag ctc-3'	204 bp	*trxA*	F: 5'-gtg gcc ggc acc ctg aag cat gtg-3' R: 5'-tca gtc ggc gat gaa gtc ctc gag-3'	333 bp
*dnaK*	F: 5'-cgc cag gcg gtc acg aac gtc gac-3' R: 5'-ctg gaa ctg ctt gac cag gta gtc-3'	480 bp	*trxA2*	F: 5'-gtg ccc gag gtg acg gac gcg-3' R: 5'-tca gac cac gtc ggc cag ctc-3'	315 bp
*dnaJ*	F: 5'-gac ggt gag gtt gtc gcc ccg gcg-3' R: 5'-cgc acc ggc ggc gga ccg ggc acc-3'	504 bp	*trxA3*	F: 5'-atg acc agc acc gtg gaa ctc acc-3' R: 5'-cta ctg gcc ttc ctg gcc ttc ctg-3'	405 bp
*grpE*	F: 5'-ccg cgg acc tcc agc ggc tcc agg-3' R: 5'-cct tgg tcc cgg cgt cgt cct ccg-3'	474 bp	*trxB*	F: 5'-ctc tac acc gcg cga gca tcc ctg-3' R: 5'-ctt cag gcc cgc aag ctt ctg gtc-3'	594 bp
*groEL2*	F: 5'-gac gac gtc gcc ggt gac ggt acg-3' R: 5'-cga cag ggc ctc gcc ctc gac gtc-3'	534 bp	*trxB2*	F: 5'-ggc ggt tcg ctc acg acc acg-3' R: 5'-ctc gcc ggt cag ggt gtc acg-3'	563 bp

### Proteomic analysis

Cells were suspended in TE buffer containing COCKTAIL (Roche), and then disrupted with a cell homogenizer. Samples containing 100 μg proteins were prepared from the supernatant for the subsequent 1D- and 2D-analyses. For 1D-LC/ESI-MS/MS analysis, protein samples were treated by 1D-SDS-PAGE. Protein bands were excised and digested in-gel with trypsin. To identify the major proteins, tryptic peptides were submitted for Electrospray Ionization Quadrupole-Time of Flight instrument (ESI-Q-TOF MS). Peptide mass fingerprints were analyzed by using the MASCOT . In 2D-gel electrophoresis, 18 cm IPG strips pH 4~7 (Amersham Biosciences) were used for the first-dimension isoelectric focusing. Gels were stained with a silver staining kit (Amersham Biosciences) and scanned by using a UMAX power look 1120 scanner (UMAX, Taiwan). Image analysis was performed by using Phoretix 2D Expression (NonLinear Dynamics, UK). Protein identification was performed by using a Matrix Assisted Laser Desorption/Ionizing-Time of Flight (MALDI-TOF) mass spectrometry system (Voyager DE-STR, PE Biosystem, Framingham, MA) in NICEM (National Instrumentation Center for Environmental Management). Other experimental conditions were detailed in our previous report [10].

## Authors' contributions

YJK conceived of the study, and participated in its design and coordination. YJK, MHM, and JYS performed the experiments. YKC supervised the study and prepared the manuscript with YJK. CPS and SKH guided YJK's analysis of DNA chip and proteomic data. All authors read and approved the final manuscript.

## References

[B1] Rehm HJ, Reed G (1997). Biotechnology: products of secondary metabolism.

[B2] Hayes A, Hobbs G, Smith CP, Oliver SG, Butler PR (1997). Environmental Signals Triggering Methylenomycin Production by *Streptomyces coelicolor *A3(2). J Bacteriol.

[B3] Sevcikova B, Kormanec J (2004). Differential prodcution of two antibiotics of *Streptomyces coelicolor *A3(2), actinorhodin and undecylprodigiosin, upon salt stress conditions. Arch Microbiol.

[B4] Mikilik K, Khanh-Hoang Q, Halada P, Bezoouskova S, Benada O, Behal V (1999). Expression of the Csp protein family upon cold shock and production of tetracycline in *Streptomyces aureofaciens*. Biochem Biophysic Res Comm.

[B5] Kim CJ, Chang YK, Chun GT (2000). Enhancement of Kasugamycin Production by the pH Shock in Batch Cultures of *Streptomyces kasugaensis*. Biotechnol Prog.

[B6] Kim CJ, Chang YK, Chun GT, Jeong YH, Lee SJ (2001). Continuous Culture of Immobilized *Streptomyces *Cells for Kasugamycin Production. Biotechnol Prog.

[B7] Vohradsky J, Li XM, Dale G, Folcher M, Nguyen L, Viollier PH, Thompson CJ (2000). Developmental Control of Stress Stimulons in *Streptomyces coelicolor *Revealed by Statistical Analyses of Global Gene Expression Patterns. J Bacteriol.

[B8] Kelemen GH, Viollier PH, Tenor JL, Marri L, Buttner MJ, Thompson CJ (2001). A connection between stress and development in the multicellular prokaryote *Streptomyces coelicolor *A3(2). Mol Microbiol.

[B9] Novotna J, Vohradsky J, Berndt P, Gramajo H, Langen H, Li XM, Minas W, Orsaria L, Roeder D, Thompson CJ (2003). Proteomic studies of diauxic lag in the differentiating prokaryote *Streptomyces coelicolor *reveal a regulatory network of stress-induced proteins and central metabolic enzymes. Mol Microbiol.

[B10] Kim YJ, Song JY, Moon MH, Smith CP, Hong SK, Chang YK (2007). pH shock induces overexpression of regulatory and biosynthetic genes for actinorhodin production in *Streptomyces coelicolor *A3(2). Appl Microbiol Biotechnol.

[B11] Arias P, FernÃ¡ndez-Moreno M, Malpartida F (1999). Characterization of the pathway-specific positive transcriptional regulator for actinorhodin biosynthesis in *Streptomyces coelicolor *A3(2) as a DNA-binding protein. J Bacteriol.

[B12] Colombo V, FernÃ¡ndez-de-Heredia M, Malpartida F (2001). A polyketide biosynthetic gene cluster from Streptomyces antibioticus includes a LysR-type transcriptional regulator. Microbiology.

[B13] Kim ES, Cramer KD, Shreve AL, Sherman DH (1995). Heterologous expression of an engineered biosynthetic pathway: functional dissection of type II polyketide synthase components in *Streptomyces *species. J Bacteriol.

[B14] Sherman DH, Kim ES, Bibb MJ, Hopwood DA (1992). Functional replacement of genes for individual polyketide synthase components in *Streptomyces coelicolor *A3(2) by heterologous genes from a different polyketide pathway. J Bacteriol.

[B15] Fernández-Moreno MA, Martinez E, Cabalero JL, Ichinose K, Hopwood DA, Malpartida F (1994). DNA Sequence and Function of the actVI Region of the Actinorhodin Biosynthetic Gene Cluster of *Streptomyces coelicolor *A3(2). J Biol Chem.

[B16] Ichinose K, Surti C, Taguchi T, Malpartida F, Booker-Milburn KI, Stephenson GR, Ebizuka Y, Hopwood DA (1999). Proof that the ACTVI genetic region of *Streptomyces coelicolor *A3(2) is involved in stereospecific pyran ring formation in the biosynthesis of actinorhodin. Bioorg Med Chem Lett.

[B17] Ichinose K, Taguchi T, Bedford DJ, Ebizuka Y, Hopwood DA (2001). Functional complementation of pyran ring formation in actinorhodin biosynthesis in *Streptomyces coelicolor *A3(2) by ketoreductase genes for granaticin biosynthesis. J Bacteriol.

[B18] Caballero JL, Martinez E, Malpartida F, Hopwood DA (1991). Organization and functions of the actVA region of the actinorhodin biosynthetic gene cluster of *Streptomyces coelicolor*. Mol Gen Genet.

[B19] Seveikova B, Benada O, Kofronova O, Kormanec J (2001). Stress-response sigma factor σ^H ^is essential for morphological differentiation of *Streptomyces coelicolor *A3(2). Arch Micobiol.

[B20] Lee EJ, Karoonuthaisiri N, Kim HS, Park JH, Cha CJ, Kao CM, Roe JH (2005). A master regulator σ^B ^governs osmotic and oxidative response as well as differentiation via a network of sigma factors in *Streptomyces coelicolor*. Mol Microbiol.

[B21] Cho YH, Lee EJ, Ahn BE, Roe JH (2001). SigB, an RNA polymerase sigma factor required for osmoprotection and proper differentiation of *Streptomyces coelicolor*. Mol Microbiol.

[B22] Bae JB, Park JH, Hahn MY, Kim MS, Roe JH (2004). Redox-dependent changes in RsrA, an anti-sigma factor in *Streptomyces coelicolor *: zinc release and disulfide bond formation. J Mol Biol.

[B23] Takano H, Hosono K, Beppu T, Ueda K (2003). Involvement of sigma (H) and related sigma factors in glucose-dependent initiation of morphological and physiological development of *Streptomyces griseus*. Gene.

[B24] Kormanec J, Sevcikova B, Halgasova N, Knirschova R, Rezuchova B (2000). Identification and transcriptional characterization of the gene encoding the stress-response sigma factor sigma (H) in *Streptomyces coelicolor *A3(2). FEMS Microbiol Lett.

[B25] Marcos AT, Gutierrez S, Diez B, Fernandez FJ, Oguiza JA, Martin JF (3570). Three genes hrdB, hrdD and hrdT of *Streptomyces griseus *IMRU encoding sigma factor-like proteins, are differentially expressed under specific nutritional conditions. Gene.

[B26] Kang JG, Hahn MY, Akira I, Roe JH (1997). Identification of sigma factors for growth phase-related promoter selectivity of RNA polymerases from *Streptomyces coelicolor *A3(2). Nucleic Acids Research.

[B27] Fujii T, Gramajo HC, Takano E, Bibb MJ (1996). *RedD *and *actII*-ORF4, pathway-specific regulatory genes for antibiotic production in *Streptomyces coelicolor *A3(2), are transcribed in vitro by an RNA polymerase holoenzyme containing sigma *hrdD*. J bacteriol.

[B28] Karoonuthaisiri N, Weaver D, Huang J, Cohen SN, Kao CM (2005). Regional organization of gene expression in *Streptomyces coelicolor*. Gene.

[B29] Conway de Macario E, Dugan CB, Macario AJ (1994). Identification of a grpE heat-shock gene homolog in the archaeon *Methanosarcina mazei*. J Mol Biol.

[B30] Bucca G, Brassington AM, Hotchkiss G, Mersinias V, Smith CP (2003). Negative feedback regulation of dnaK, clpB and Ion expression by the DnaK chaperone machine in *Streptomyces coelicolor*, identified by transcriptome and in vivo DnaK-depletion analysis. Mol Microbiol.

[B31] Bucca G, Ferina G, Puglia AM, Smith CP (1995). The dna K operon of *Streptomyces coelicolor *encodes a novel heat-shock protein shich binds to the promoter region of the operon. Mol Microbiol.

[B32] Barreiro C, Gonzalex-Lavado E, Patek M, Martin JF (2004). Transcriptional Analysis of the GroES-groEL1, groEL2 and dnaK genes in *Corynebacterium glutamicum *: Characterization of Heat Shock-Induced Promoters. J Bacteriol.

[B33] Duchene AM, Thompson CJ, Mazodier P (1994). Transcriptional analysis of groEL genes in *Streptomyces coelicolor *A3(2). Mol Gen Genet.

[B34] Guglielmi G, Mazodier P, Thompson CJ, Davies J (1991). A Survey of the Heat Shock Response in Four *Streptomyces *Species Reveals Two groEL-Like Genes and Three GroEL-Like Proteins in *Streptomyces albus*. J Bacteriol.

[B35] Chung HJ, Choi JH, Kim EJ, Cho YH, Roe JH (1999). Negative regulation of the gene for Fe-containing superoxide dismutase by an Ni-responsive factor in *Streptomyces coelicolor*. J Bacteriol.

[B36] Chung HJ, Kim EJ, Suh B, Choi JH, Roe JH (1999). Duplicate genes for Fe-containing superoxide dismutase in *Streptomyces coelicolor *A3(2). Gene.

[B37] Cho YH, Lee EJ, Roe JH (2000). A developmentally regulated catalase required for proper differentiation and osmoprotection of *Streptomyces coelicolor*. Mol Microbiol.

[B38] Kang JG, Paget MS, Seok YJ, Hahn MY, Bae JB, Hahn JS, Kleanthous C, Buttner MJ, Roe JH (1999). RsrA, an anti-sigma factor regulated by redox change. EMBO J.

[B39] Paget MS, Kang JG, Roe JH, Buttner MJ (1998). Sigma R, an RNA polymerase sigma factor that modulates expression of the thioredoxin system I response to oxidative stress in *Streptomyces coelicolor *A3(2). EMBO J.

[B40] Paget MS, Molle V, Cohen G, Aharonowitz Y, Buttner MJ (2001). Defining the disulphide stress response in *Streptomyces coelicolor *A3(2): identification of the sigmaR regulon. Mol Microbiol.

[B41] Kieser T, Bibb MJ, Buttner MJ, Chater KF, Hopwood DA (2000). Practical Streptomyces genetics.

[B42] Bystrykh LV, Fernández-Moreno MA, Herrema JK, Malpartida F, Hopwood DA, Dijkhuizen L (1996). Production of Actinorhodin-Related "Blue Pigments" by *Streptomyces coelicolor *A3(2). J Bacteriol.

